# Subtherapeutic posaconazole exposure during delayed-release tablet prophylaxis in high-risk patients with haematological malignancies: rationale for routine therapeutic drug monitoring

**DOI:** 10.1093/jac/dkag233

**Published:** 2026-07-15

**Authors:** Karen F Urbancic, Michelle K Yong, KarYee Yong, Hayley Page, Marcelle Stewart, David Ritchie, Ashish Bajel, Eric Wong, Chun Fong, Jason A Trubiano, David C M Kong, Monica A Slavin

**Affiliations:** Pharmacy Department, Austin Health, Heidelberg, VIC, Australia; Department of Infectious Diseases and Immunology, Austin Health, Heidelberg, VIC, Australia; National Centre for Infections in Cancer, National Health and Medical Research Council Centre of Research Excellence, Peter MacCallum Cancer Centre, Department of Oncology, University of Melbourne, Parkville, VIC, Australia; Department of Medicine, University of Melbourne, Parkville, VIC, Australia; National Centre for Infections in Cancer, National Health and Medical Research Council Centre of Research Excellence, Peter MacCallum Cancer Centre, Department of Oncology, University of Melbourne, Parkville, VIC, Australia; Department of Medicine, University of Melbourne, Parkville, VIC, Australia; Infectious Diseases Unit, Peter MacCallum Cance Centre, Parkville, VIC, Australia; Sir Peter MacCallum Department of Oncology, The University of Melbourne, Parkville, VIC, Australia; National Centre for Infections in Cancer, National Health and Medical Research Council Centre of Research Excellence, Peter MacCallum Cancer Centre, Department of Oncology, University of Melbourne, Parkville, VIC, Australia; Infectious Diseases Unit, Peter MacCallum Cance Centre, Parkville, VIC, Australia; National Centre for Infections in Cancer, National Health and Medical Research Council Centre of Research Excellence, Peter MacCallum Cancer Centre, Department of Oncology, University of Melbourne, Parkville, VIC, Australia; Infectious Diseases Unit, Peter MacCallum Cance Centre, Parkville, VIC, Australia; National Centre for Infections in Cancer, National Health and Medical Research Council Centre of Research Excellence, Peter MacCallum Cancer Centre, Department of Oncology, University of Melbourne, Parkville, VIC, Australia; Infectious Diseases Unit, Peter MacCallum Cance Centre, Parkville, VIC, Australia; Clinical Haematology Department, Peter MacCallum Cancer Centre and the Royal Melbourne Hospital, Melbourne, VIC, Australia; Department of Medicine, University of Melbourne, Parkville, VIC, Australia; Clinical Haematology Department, Peter MacCallum Cancer Centre and the Royal Melbourne Hospital, Melbourne, VIC, Australia; Department of Medicine, University of Melbourne, Parkville, VIC, Australia; Clinical Haematology Department, Austin Health, Heidelberg, VIC, Australia; Clinical Haematology Department, Austin Health, Heidelberg, VIC, Australia; Department of Infectious Diseases and Immunology, Austin Health, Heidelberg, VIC, Australia; Department of Infectious Diseases, University of Melbourne, the Peter Doherty Institute for Immunity and Infection, Melbourne, VIC, Australia; Centre for Medicine Use and Safety, Monash University, Parkville, VIC, Australia; National Centre for Infections in Cancer, National Health and Medical Research Council Centre of Research Excellence, Peter MacCallum Cancer Centre, Department of Oncology, University of Melbourne, Parkville, VIC, Australia; Department of Medicine, University of Melbourne, Parkville, VIC, Australia; Infectious Diseases Unit, Peter MacCallum Cance Centre, Parkville, VIC, Australia; Victorian Infectious Diseases Service, The Peter Doherty Institute for Immunity and Infection, Royal Melbourne Hospital, Melbourne, Victoria, Australia

## Abstract

**Background:**

Posaconazole prophylaxis is indicated in high-risk patients with haematological malignancies to prevent invasive fungal diseases (IFDs) with guidelines advising steady-state posaconazole plasma concentrations (PPCs) above 0.5–0.7 mg/L. Therapeutic drug monitoring (TDM), however, is not routinely recommended for posaconazole delayed-release tablet (DRT) prophylaxis.

**Objectives:**

To describe PPCs in hospitalized high-risk patients with haematological malignancies treated for AML or undergoing allogeneic haematopoietic cell transplantation receiving posaconazole prophylaxis with posaconazole DRT and factors influencing exposure.

**Patients and methods:**

This prospective, two-centre cohort study measured serial PPCs at Days 7, 14 and 21, and during diarrhoea episodes in adult high-risk patients receiving prophylaxis with posaconazole DRT between August 2019 and May 2023. Patients were followed during hospital admission and for 7 days after the last dose of posaconazole or hospital discharge.

**Results:**

Ninety-two patients contributed 223 PPCs. Subtherapeutic PPCs (<0.7 mg/L) occurred in 77 (34.5%) samples and 49 (53.3%) patients recorded ≥1 subtherapeutic PPC. The median Day 7 PPC was 0.84 (IQR: 0.47–1.16) mg/L, with no significant changes over time. In patients with diarrhoea compared with no diarrhoea, the median PPC was significantly lower [0.74 (IQR: 0.48–1.00) mg/L versus 0.92 (IQR: 0.64–1.40) mg/L, *P* = 0.007]. Multivariate analysis identified older age (>60 years) was protective against subtherapeutic PPCs. Six IFDs developed in five patients (5.4%) during follow-up and posaconazole-attributed hepatotoxicity resulted in cessation for one patient (1.1%).

**Conclusions:**

Subtherapeutic PPCs are common during posaconazole DRT prophylaxis, suggesting the need for routine TDM to optimize dosing in those receiving this posaconazole formulation.

## Introduction

Patients receiving chemotherapy for AML and myelodysplastic neoplasm (MDS), as well as allogeneic haematopoietic cell transplant (alloHCT) recipients, are at high risk of developing invasive fungal diseases (IFDs) that contribute significantly to mortality and morbidity.^1–3^ Posaconazole, a broad spectrum triazole antifungal agent, has demonstrated efficacy in preventing IFDs and improving survival in these high-risk patients.^4,5^ Although debate exists about the optimal target pre-dose posaconazole plasma concentration (PPC) for prophylaxis efficacy,^6^ an exposure–response relationship has been established in the clinical setting^7,8^ with studies suggesting pre-dose PPCs <0.5 or <0.7 mg/L are associated with an increased risk of breakthrough IFD.^7,9–16^ Current consensus guidelines recommended therapeutic drug monitoring (TDM) as a tool to identify poor posaconazole absorbers with a target of either ≥0.5^17,18^ or ≥0.7 mg/L for prophylaxis,^19,20^ although there is debate as to whether PPCs should be performed routinely in patients receiving the delayed-release oral tablet (DRT) formulation.^21^ This formulation has been reported to have less variable bioavailability and >90% achievement of target PPCs^22,23^ compared with the oral suspension. In addition, the impact of compromised gastrointestinal (GI) function including diarrhoea on PPCs achieved with this formulation has been explored and identified as a potential risk factor for lower PPCs.^24–29^ So far, however, these data remain limited largely to retrospective analyses describing the impact of diarrhoea on initial steady-state PPCs.^24–29^ Therefore, the primary aim of this study was to investigate using a prospective study design, the pre-dose PPCs achieved throughout a course in patients receiving posaconazole DRT prophylaxis. We also aimed to identify risk factors associated with subtherapeutic PPCs, including markers of impaired GI absorption, on achievement of therapeutic targets.

## Patients and methods

### Study design and population

This prospective, two-centre, observational study was performed in hospitalized patients with haematological malignancies receiving posaconazole DRT prophylaxis at two centres in Melbourne, Australia [Royal Melbourne (RMH) and Austin Hospitals (AH)] between August 2019 and May 2023. These are university-affiliated tertiary hospitals with large adult haematology services managing complex patients with acute leukaemia and perform alloHCT. Adult patients with haematological malignancies receiving posaconazole DRT prophylaxis in the inpatient setting as standard of care were assessed for inclusion in the study. Patients were excluded if they were receiving secondary posaconazole prophylaxis for proven, probable or possible IFD, or if posaconazole DRT was switched to intravenous (IV) posaconazole or an alternative antifungal agent before the initial PPC sample being performed. Participants were followed with respect to clinical data collection during the PPC sample collection period. Follow-up for breakthrough IFD was conducted for 7 days following the last dose of posaconazole for inpatients or for 7 days following hospital discharge. Patients diagnosed with IFD were subsequently followed for 30 days after diagnosis to evaluate clinical outcomes.

### Ethics

Written informed consent was obtained from all participants at study entry. Ethics approval was obtained from the human research ethics committee (approval number HREC/17/MH/349).

### Local antifungal prophylaxis policies

High-risk patients with haematological malignancies requiring anti-mould prophylaxis received posaconazole according to hospital protocols that were similar at both hospitals and based on the Australasian consensus guidelines.^30^ This included predominantly patients with AML/MDS receiving intensive chemotherapy and alloHCT recipients during periods of prolonged neutropenia or graft-versus-host-disease (GvHD) requiring intensive immunosuppression. Posaconazole DRT formulation was initiated usually at 300 mg once daily. Loading doses of 300 mg orally twice daily on the first day were given according to clinician preference. Duration of prophylaxis followed local protocols: patients with AML/MDS continued oral posaconazole prophylaxis until neutrophil recovery; alloHCT recipients continued for approximately 3 months post-transplant or, in the case of GvHD requiring immunosuppression, at least 16 weeks or until immunosuppression was tapered, whichever was later. IV posaconazole prophylaxis was used if subtherapeutic PPCs were suspected or proved, or oral administration was not tolerated, and alternative antifungals were considered if IFD or posaconazole toxicity occurred. At RMH, PPCs were not routinely measured or reported in real-time. At AH, PPCs were performed routinely at steady state, then weekly or more often if malabsorption was suspected. PPC results continued to be reported to the treating clinicians on their availability from the referral laboratory to inform clinical decisions such as oral dose escalation, IV switch and empiric antifungal treatment decisions. The recommended target PPC was ≥0.5 mg/L.^17^

### Data collection and definitions

Prospective data were collected including: patient demographics, clinical data and outcomes were extracted from the electronic medical record including clinical notes, medication records, pathology and imaging. PPCs were performed at both sites at steady state on Days 7, 14 and 21 ± 1 day of DRT prophylaxis. Further PPCs were collected 48–72 hours after diarrhoea onset, then twice weekly up to Day 21, and before switch to antifungal treatment if IFD was suspected. Concurrent clinical observations and liver function tests (LFTs) were recorded before commencement of posaconazole DRT prophylaxis, and within 48 hours of each PPC sampling to assess potential factors affecting posaconazole exposure. These included interacting medications including proton pump inhibitors (PPIs), total parenteral nutrition (TPN), oral mucositis, diarrhoea, vomiting, posaconazole dose and active GI GvHD according to Common Terminology Criteria for Adverse Events (CTCAE) v.5.0 grading where relevant.^31^ IFDs were assessed on the basis of international definitions of proven, probable or possible IFD^32^ in patients who underwent IFD-related investigations and/or commenced antifungal treatment during the study period and for up to 7 days after the last dose of posaconazole for inpatients or for 7 days following hospital discharge. Breakthrough IFD was defined as proven, probable or possible IFD occurring while the patient was receiving posaconazole prophylaxis, consistent with established definitions in the literature.^33^ Subtherapeutic PPCs were defined as <0.7 mg/L according to clinical studies and guidelines.^12,17,19,20^ The threshold <0.5 mg/L was also evaluated.^11^ As no definitive toxicity threshold exists, 3.75 mg/L was used as a reference limit based on DRT formulation pharmacokinetic studies.^22^

### Laboratory methods and posaconazole assay

PPC analysis was conducted at a referral laboratory using an ultra-performance liquid chromatography tandem mass spectrometric method in-house assay (high-performance LC–tandem MS, Waters Acquity system).^34^ Samples were centrifuged and extracted plasma stored at 4°C or frozen until analysis in accordance with established laboratory procedures.

### Primary outcomes

Two primary outcomes were evaluated: (i) number and proportion of patients with subtherapeutic pre-dose PPCs during the study and (ii) median and IQR pre-dose PPC overall, initially (i.e. Day 7), and across therapy (i.e. Days 14 and 21).

### Secondary outcomes

Secondary outcomes included: (i) patient and clinical risk factors associated with subtherapeutic PPCs; (ii) frequency of posaconazole-attributed toxicities resulting in discontinuation, including liver function derangement according to CTCAE v.5.0 grading^31^; (iii) incidence of proven, probable or possible IFD^32^ including fungal epidemiology and PPCs before diagnosis and (iv) impact of oral posaconazole dose escalation on achievement of therapeutic PPCs.

### Statistical analysis

Patient characteristics and outcomes were reported using descriptive statistics. PPCs were summarized using medians and IQRs. Proportion of subtherapeutic PPCs (both <0.5 and <0.7 mg/L) were calculated based on both Australasian and international guideline recommendations for prophylaxis thresholds.^18–20^ Categorical data were compared using Fisher’s exact or chi-square tests. Continuous variables were compared by Mann–Whitney *U*-test or Kruskal–Wallis tests as appropriate. Inter-patient variability was calculated as the coefficient of variation (CV) of mean PPCs, while intra-patient variability was assessed as the CV of PPCs in participants with repeated samples. Generalized estimating equation logistic regression was used to identify independent risk factors for subtherapeutic PPCs while accounting for repeated measurements per patient. The analysis included 88 patients (218 PPCs) with AML/MDS or alloHCT recipients. Patients receiving posaconazole prophylaxis for other indications (*n* = 4; five PPCs) were excluded from the multivariate analysis due to patient heterogeneity. Candidate variables for multivariate analysis were selected from univariate analysis (*P* < 0.20) and/or excluded if insufficient events were captured to be clinically meaningful. All analyses were performed using R version 4.3.1, where a *P* value <0.05 was considered statistically significant.

## Results

### Baseline patient characteristics

Baseline patient characteristics are summarized in Table 1. Of the 92 patients with PPCs, 63 (68.5%) were male with a median age of 58 (IQR 45–65) years and age-adjusted Charlson Comorbidity Score of 3 (IQR 2–4). The most common diagnosis was AML (44/92, 47.8%). The primary indications for posaconazole prophylaxis were either pre-engraftment alloHCT (51/92, 55.4%) or AML/MDS receiving chemotherapy (37/92, 40.2%).

**Table 1.** Patient demographics and transplant conditions

Demographics	Overall (*n* = 92)	Indication for posaconazole		
		AlloHCT (*n* = 51)	AML/MDS (*n* = 37)	Other^a^ (*n* = 4)
Age (years), median (IQR)	58 (45–65)	60 (49–66)	54 (41–63)	70 (68–71)
Gender				
Male, *n* (%)	63 (68)	37 (73)	24 (65)	2 (50)
Female, *n* (%)	29 (32)	14 (27)	13 (35)	2 (50)
Ethnicity				
Caucasian, *n* (%)	77 (84)	42 (82)	31 (84)	4 (100)
Asian, *n* (%)	6 (6.5)	4 (7.8)	2 (5.4)	0 (0)
African, *n* (%)	2 (2.2)	4 (7.8)	2 (5.4)	0 (0)
Hispanic, *n* (%)	1 (1.1)	0 (0)	2 (5.4)	0 (0)
Other, *n* (%)	6 (6.5)	1 (2.0)	0 (0)	0 (0)
Charlson Comorbidity Index, median (IQR)	3 (2, 4)	3 (2, 4)	4 (2, 4)	4.5 (4, 5)
Malignancy				
AML, *n* (%)	44 (48)	8 (16)	36 (97)	0 (0)
Lymphoma^b^, *n* (%)	20 (22)	18 (35)	0 (0)	2 (50)
Myelofibrosis, *n* (%)	6 (6.5)	6 (12)	0 (0)	0 (0)
Acute lymphoblastic leukaemia, *n* (%)	5 (5.4)	5 (9.8)	0 (0)	0 (0)
MDS, *n* (%)	5 (5.4)	5 (9.8)	0 (0)	0 (0)
Chronic lymphocytic leukaemia, *n* (%)	3 (3.3)	3 (5.9)	0 (0)	0 (0)
Chronic myeloid leukaemia, *n* (%)	3 (3.3)	2 (3.9)	1 (2.7)	0 (0)
Chronic myelomonocytic leukaemia, *n* (%)	2 (2.2)	2 (3.9)	0 (0)	0 (0)
Multiple myeloma, *n* (%)	2 (2.2)	0 (0)	0 (0)	2 (50)
Aplastic anaemia, *n* (%)	1 (1.1)	1 (2.0)	0 (0)	0 (0)
T-cell prolymphocytic leukaemia, *n* (%)	1 (1.1)	1 (2.0)	0 (0)	0 (0)
AlloHCT				
Receipt of alloHCT, *n* (%)	51 (55)	51 (100)	—	—
Conditioning intensity				
Reduced intensity conditioning, *n* (%)	41 (80)	41 (80)	—	—
Myeloablative conditioning, *n* (%)	10 (20)	10 (20)	—	—
T-cell depletion				
Antithymocyte globulin, *n* (%)	26 (51)	26 (51)	—	—
None, *n* (%)	24 (47)	24 (47)	—	—
Alemtuzumab, *n* (%)	1 (2.0%)	1 (2.0%)	—	—

^a^Other indications for posaconazole include: autologous HCT (*n* = 3); chimeric antigen receptor T-cell therapy (*n* = 1)

^b^Lymphoma includes either non-Hodgkin or Hodgkin lymphoma diagnoses

**Figure 1.**
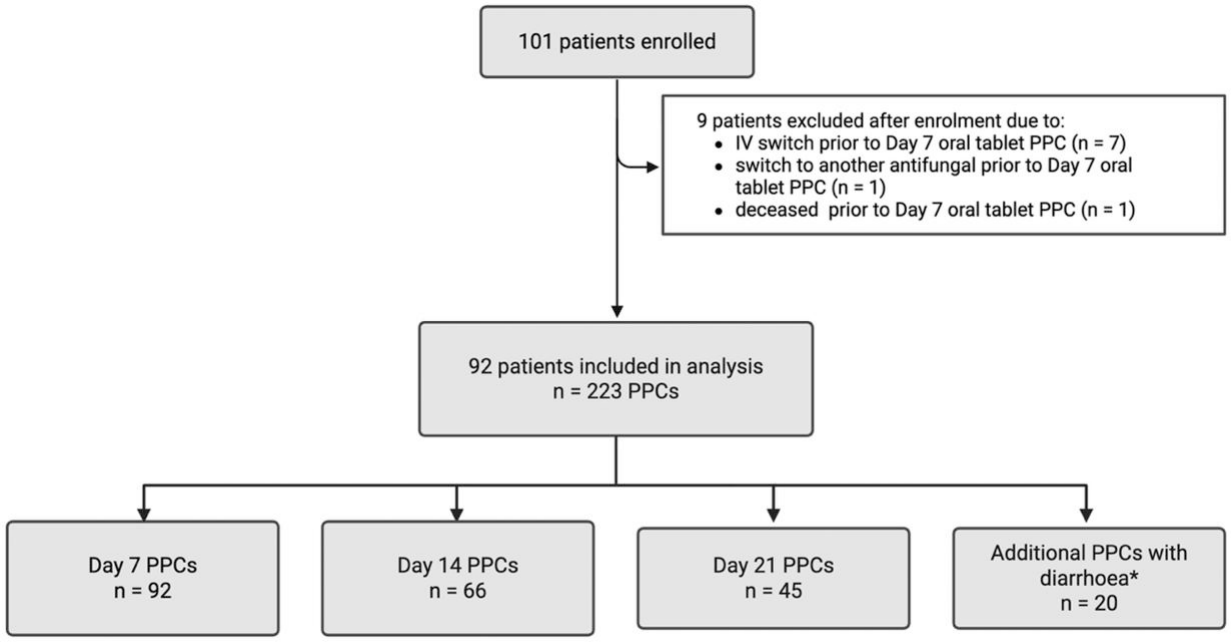
Study enrolment and number of PPCs collected at pre-specified timepoints. *Taken at other time points during the 21-day study period.

Figure 1 summarizes primary enrolment, reasons for excluded patients and PPC collection details at pre-specified timepoints. The primary reason for patient dropout at each PPC timepoint after Day 7 was due to switch to IV posaconazole formulation (31/92, 33.7%), with 61 patients (66.3%) remaining on DRT formulation during the study period. The median duration of posaconazole DRT prophylaxis during inpatient admission was 20 days (IQR 12–28 days).

### Primary outcomes

**Figure 2.**
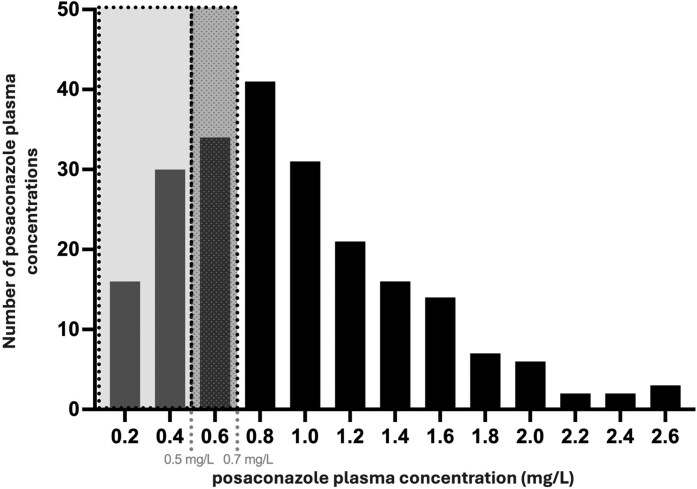
Distribution of all PPCs recorded (*n* = 223). Overall, 77 out of 223 (34.5%) PPCs were <0.7 mg/L and 46 (20.6%) PPCs were <0.5 mg/L.

Within this period, 223 pre-dose PPCs were measured: 92 at Day 7; 66 at Day 14 and 45 at Day 21, with an additional 20 pre-dose PPCs measured during significant diarrhoea (Figure 1). A median of 3 (IQR: 2,3) inpatient pre-dose PPCs were performed per patient. Overall, 49/92 patients (53.2%) recorded ≥1 PPC <0.7 mg/L, with 77/223 (34.5%) of pre-dose PPCs <0.7 mg/L. Thirty-two of 92 patients (34.8%) recorded ≥1 PPC <0.5 mg/L with 46/223 (20.6%) PPCs under this threshold (Figure 2). No PPCs were >3.75 mg/L.

**Figure 3.**
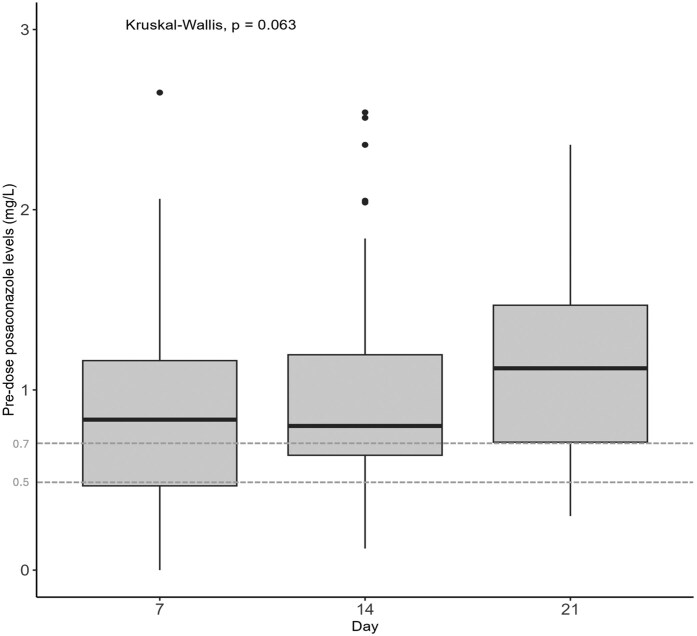
PPCs at serial timepoints during prophylaxis. Box plots showing pre-dose PPCs at Days 7, 14 and 21 of DRT prophylaxis. The horizontal line within each box represents the median, boxes indicate the IQR, whiskers represent 1.5 × IQR and dots denote outliers. Dashed horizontal lines indicate the prophylactic targets of 0.7 and 0.5 mg/L.

The median (IQR) pre-dose PPC overall was 0.83 (0.55–1.24) mg/L. Figure 3 shows the distribution of PPCs measured throughout posaconazole DRT prophylaxis, with no statistically significant changes identified across the course (Kruskal–Wallis rank sum test; *P* = 0.063). The median PPC (IQR) at each timepoint were: Day 7 [0.84 (IQR: 0.47–1.16)], Day 14 [0.80 (0.64–1.20)] and Day 21 [1.12 (0.71–1.47)] mg/L.

For inter-patient variability, the CV was 51% for the 92 patients. For intra-patient variability, 72 patients were included and yielded a CV of 23%.

### Secondary outcomes

Unadjusted comparison of the median PPCs in the presence and absence of any grade diarrhoea found a statistically significant difference [0.74 (IQR: 0.48–1.00) mg/L versus 0.92 (0.64–1.40) mg/L, *P* = 0.007]. Table 2 summarizes results from the univariate and multivariate analyses of patient and clinical risk factors for PPCs <0.7 and <0.5 mg/L. The analysis included 88 patients with AML/MDS or alloHCT recipients (218 PPCs) while patients receiving posaconazole prophylaxis for other indications (four patients; five PPCs) were excluded from this part of the analysis. Age >60 years old was identified as being associated with lower odds of PPCs <0.7 and <0.5 mg/L on multivariate analysis, respectively (adjusted OR = 0.31, 95% CI: 0.12–0.78, *P* = 0.013; adjusted OR = 0.14, 95% CI: 0.04–0.48, *P* = 0.002). No other clinical covariates were identified as independently influencing PPCs achieved in this patient cohort including diarrhoea for either threshold. No patients in our study received concurrent interacting medications known to affect reduce posaconazole clearance such as rifampicin or phenytoin.

**Table 2.** Univariate and multivariate analysis of patient-related and clinical risk factors associated with subtherapeutic PPCs (<0.7 and <0.5 mg/L)

	POS plasma concentrations *n* = 223				GEE logistic regression (0.7 mg/L threshold)						GEE logistic regression (0.5 mg/L threshold)					
Variable(s)					Univariable			Multivariable			Univariable			Multivariable		
	<0.7 mg/L *n* = 77 (34.5%)	≥0.7 mg/L *n* = 146 (65.5%)	<0.5 mg/L *n* = 46 (20.6%)	≥0.5 mg/L *n* = 177 (79.4%)	OR	(95%CI)	*P*	OR	(95%CI)	*P*	OR	(95%CI)	*P*	OR	(95%CI)	*P*
Age group (years)																
<50	28 (36%)	39 (27%)	18 (39%)	49 (28%)	REF	REF	REF	REF	REF	REF	REF	REF	REF	REF	REF	REF
50–59	26 (34%)	25 (17%)	19 (41%)	32 (18%)	1.21	0.49–0.031	0.663	1.38	0.51–3.73	0.508	1.25	0.49–3.21	0.647	1.34	0.53–3.40	0.566
>60	23 (30%)	82 (56%)	9 (20%)	96 (54%)	0.36	0.16–0.81	**0**.**020**	0.31	0.12-0.78	**0.013**	0.21	0.08–0.59	**0**.**007**	0.14	0.04–0.48	**0.002**
Weight (kg)	84 (76–100)	81 (71–91)	85 (74–98)	81 (75–92)	1.010	0.99–1.03	0.201	—	—	—	1.010	0.99–1.03	0.404	—	—	—
Gender																
Female	54 (70%)	104 (71%)	33 (72%)	125 (71%)	REF	REF	REF	—	—	—	REF	REF	REF	—	—	—
Male	23 (30%)	42 (29%)	13 (28%)	52 (29%)	0.920	0.49–1.73	0.768	—	—	—	1.040	0.52–2.08	0.929	—	—	—
Indication for POS																
AML/MDS	31 (40%)	74 (51%)	14 (30%)	92 (51%)	REF	REF	REF	REF	REF	REF	REF	REF	REF	REF	REF	REF
AlloHCT	44 (57%)	69 (47%)	30 (65%)	83 (47%)	0.620	0.30–1.28	**0**.**195**	1.09	0.49–2.44	0.836	0.410	0.17–0.96	**0**.**048**	1.89	0.77–4.62	0.181
Other^a^	2 (2.6%)	3 (2.1%)	2 (4.3%)	3 (1.7%)	—	—	—	—	—	—	—	—	—	—	—	**—**
PPI use	67 (89%)	128 (88%)	42 (95%)	153 (86%)	1.050	0.42–2.62	0.918	—	—	—	3.090	0.75–12.68	**0**.**036**	2.77	0.58–13.38	0.088
TPN use	13 (17%)	13 (8.9%)	7 (16%)	19 (11%)	1.960	0.87–4.44	**0**.**111**	2	0.71–5.63	0.152	1.290	0.52–3.19	0.663	—	—	**—**
Diarrhoea																
No	41 (53%)	93 (64%)	23 (50%)	111 (63%)	REF	REF	REF	REF	REF	REF	REF	REF	REF	REF	REF	REF
Yes	36 (47%)	53 (36%)	23 (50%)	66 (37%)	1.480	0.86–2.53	**0**.**121**	1.26	0.65–2.46	0.384	1.470	0.81–2.65	**0**.**183**	1.12	0.55–2.29	0.736
Diarrhoea																
Grade 0	41 (53%)	93 (64%)	23 (50%)	111 (63%)	REF	REF	REF	—	—	—	REF	REF	REF	—	—	—
Grade I	17 (22%)	27 (18%)	12 (26%)	32 (18%)	1.220	0.62–2.37	0.580	—	—	—	1.360	0.67–2.79	0.440	—	—	—
Grade II	15 (19%)	19 (13%)	10 (22%)	24 (14%)	1.890	0.91–3.90	**0**.**072**	—	—	—	2.090	0.98–4.42	0.021	—	—	—
Grade III	4 (5.2%)	7 (4.8%)	1 (2.2%)	10 (5.6%)	1.590	0.50–5.05	0.419	—	—	—	0.440	0.07–2.60	0.376	—	—	—
Vomiting	2 (2.9%)	11 (7.9%)	1 (2.4%)	12 (7.1%)	0.470	0.14–1.65	**0**.**146**	—	—	—	0.440	0.10–2.02	**0**.**131**	—	—	—
Oral mucositis	19 (25%)	26 (18%)	13 (30%)	32 (18%)	1.280	0.66–2.47	0.420	—	—	—	1.350	0.66–2.79	0.497	—	—	—
Daily dose																
300 mg	73 (95%)	135 (92%)	45 (98%)	163 (92%)	REF	REF	REF	REF	REF	REF	REF	REF	REF	—	—	—
400 mg	4 (5.2%)	11 (7.5%)	1 (2.2%)	14 (7.9%)	0.230	0.05–1.08	**0**.**192**	0.18	0.14–0.92	0.119	0.11	0.01–1.80	0.376	—	—	—

GEE, generalized estimating equations; POS, posaconazole

^a^Other indications for posaconazole included: autologous HCT (*n* = 3); chimeric antigen receptor T-cell therapy (*n* = 1). These PPCs (*n* = 5) were removed from the GEE logistic regression.

One patient (1.1%) experienced grade 4 elevations in ALT and AST within 5 days of posaconazole commencement resulting in posaconazole cessation. Full recovery of LFTs and reintroduction of posaconazole without hepatotoxicity recurrence was observed. No other toxicities were identified attributable to posaconazole. Six proven or possible IFDs occurred in 5/92 patients (5.4%), with PPCs at the time of IFD diagnosis: 0.12, 0.47, 0.51, 0.76 and 2.78 mg/L. Table 3 provides clinical features of IFD cases.

**Table 3.** Invasive fungal disease cases—clinical features, epidemiology and PPCs

Patient	Age	Sex	Haematological malignancy	Allo-HCT	IFD site	Identified fungal organism	EORTC/MSG classification	Day of POS	PPC (mg/L)	Outcome (30 days after diagnosis)
1	70	Male	Chronic myelomonocytic leukemia	Yes	blood lung	*Lomentospora prolificans*	Proven	10	2.78	Died IFD related
2	66	Male	Myelodysplastic neoplasm	Yes	blood blood	*Lomentospora prolificans; Candida parapsilosis*	Proven	18	0.76	Died IFD related
3	56	Female	AML	No	blood and lung	*Lomentospora prolificans*	Proven	15	0.12	Died IFD related
4	68	Male	AML	No	blood	*Nakaseomyces glabratus*	Proven	19	0.51	Alive
5	57	Male	AML	No	lung	*Rhizopus arrhizus* (PCR positive)	Possible	22	0.47	Alive

EORTC/MSG, European Organisation for Research and Treatment of Cancer and the Mycoses Study Group; POS, posaconazole.

Of the 49 patients with a recorded subtherapeutic PPC (<0.7 mg/L), nine (18.4%) underwent oral dose escalation to 400 mg daily (given as 200 mg twice daily). Seven out of nine patients (77.8%) achieved a therapeutic PPC at steady state after dose adjustment.

## Discussion

To our knowledge, this is the first prospective, two-centre study evaluating serial PPCs in high-risk patients with haematological malignancies receiving posaconazole DRT prophylaxis. Unlike previous retrospective studies, our prospective design allowed systematic, real-time collection of clinical and pharmacokinetic data, minimizing bias and enabling more accurate assessment of temporal trends in posaconazole exposure. Despite consistent overall posaconazole exposure, 53.2% of our patients recorded at least one subtherapeutic PPC, with one-third of all PPCs being below 0.7 mg/L. This aligns with findings from retrospective, single-centre studies,^14,24,27,29,35–37^ but contrasts with earlier reports indicating >90% therapeutic PPCs.^22,23^ We observed high inter- and intra-patient variability in PPCs similar to previous studies, despite improved posaconazole exposure compared with oral suspension.^29,38,39^ Most consensus guidelines recommend only performing posaconazole TDM in those receiving posaconazole DRT prophylaxis in whom risk factors are present.^17,20,40^ However, with high prevalence of low PPCs, inter- and intra-patient variability, plus no clear independent risk factors for subtherapeutic PPCs identified in the current study, our results support routine TDM for posaconazole DRT prophylaxis. This recommendation is consistent with pharmacokinetic modelling by Lewis *et al.* showing the clinical utility for posaconazole TDM in populations when subtherapeutic exposure exceeds 10%.^41^

On multivariate analysis, we found older age (>60 years) was protective against subtherapeutic PPCs for both thresholds. Several studies have explored the relationship between age and PPCs in adults, albeit with inconsistent findings. Some evidence suggests younger age is associated with an increased risk of subtherapeutic PPCs. For instance, Tang *et al*. found that patients with PPCs <0.7 mg/L were younger (60 versus 62 years, *P* = 0.028), although this association was not statistically significant in multivariate analysis (*P* = 0.073). Similarly, Tverdek *et al*. observed a link between younger age and subtherapeutic levels (<0.7 mg/L; 48 versus 62 years, *P* = 0.03).^16^ Taken together with our findings, these results could be plausible because with younger age, receipt of more intensive chemotherapy treatment results in greater GI compromise and possible reduced absorption, and drug clearance is more efficient.

While patients who developed diarrhoea had significantly lower median PPCs, the presence of diarrhoea was not independently associated with subtherapeutic PPCs on multivariate analysis. This finding is consistent with some studies,^14,22,42,43^ although others have reported diarrhoea as a risk factor for subtherapeutic PPCs.^24,25,27,29^ These divergent results may reflect variability in the onset and severity of GI compromise that can progress beyond the first week of posaconazole and outside the timing of initial PPC measurement observed in many of these studies. In addition, in our cohort, patients with significant diarrhoea may have been switched to IV posaconazole on clinical grounds, potentially underestimating its impact on oral PPCs. Given the retrospective design and variable documentation of diarrhoea in many studies,^14,24,25,27,29,42,43^ monitoring for diarrhoea alone appears to be an unreliable surrogate for predicting subtherapeutic PPCs, supporting continued posaconazole TDM with posaconazole DRT in this population.

Furthermore, we found that neither TPN use nor concurrent PPI therapy were independent risk factors for subtherapeutic PPCs. TPN served as a surrogate for severe GI mucositis, however, this more probably reflected intolerance to oral therapy rather than low PPCs, limiting its reliability as a clinical predictor of subtherapeutic exposure. Similarly, concurrent PPI use did not affect attainment of therapeutic PPCs, consistent with previous pharmacokinetic and clinical studies that, unlike with oral suspension, acid suppression can be used concurrently with the posaconazole DRT without impact on posaconazole exposure.^15,16,24,43–45^

We did not evaluate the impact of hypoalbuminaemia in our cohort. Others have recently identified low albumin (either <35 or ≤30 g/L) as a significant predictor of subtherapeutic PPCs.^46,47^ Posaconazole is highly protein bound (>98%), and most assays measure total PPC rather than the active unbound portion. In hypoalbuminaemic patients, total PPCs may decrease, but unbound concentrations—the therapeutic component—remain stable.^48^ Therefore, total PPCs alone may underestimate effective drug levels in these patients.

Although a secondary outcome, we observed a 5.4% early IFD rate in our cohort within 28 days of commencing posaconazole DRT prophylaxis. This rate aligns with pivotal trial data and real-world studies in similar high-risk haematology cohorts.^4,5,16,49–51^ While 60% of IFD cases had subtherapeutic PPCs at the time of diagnosis (<0.7 mg/L; range 0.12–2.78 mg/L), other factors probably contributed including host immunosuppression and fungal characteristics such as intrinsic multidrug resistant, with *Lomentospora prolificans* identified in 50% of cases.

There are several limitations with our study including imprecise measurement of mucositis and diarrhoea, complicating assessment of malabsorption and possibly affecting result accuracy. Capturing the true impact of compromised GI function on PPCs is difficult because many clinicians switch to IV formulations on the basis of clinical concern rather than confirmed subtherapeutic PPCs, as evidenced in the current study by a high rate of switching to IV posaconazole. Evaluating posaconazole-attributed toxicity was challenging due to concurrent factors influencing LFTs. Our study enrolled only inpatients, potentially underrepresenting other high-risk patients receiving posaconazole as outpatients, such as those with GI GvHD on immunosuppression who are prone to malabsorption. Previous reports of lower PPCs in GvHD patients^5,14^ highlight the need for future prospective studies of posaconazole TDM to include outpatient recruitment.

In conclusion, despite the use of the DRT formulation, subtherapeutic posaconazole exposure is common in high-risk patients with haematological malignancies, which may increase vulnerability to IFD. Our results reinforce the need for routine TDM in those receiving posaconazole DRT to detect underexposure and help optimize prophylactic efficacy, especially since most patients lacked clear risk factors for subtherapeutic levels.
